# Exploring the Utility of Radiomic Feature Extraction to Improve the Diagnostic Accuracy of Cardiac Sarcoidosis Using FDG PET

**DOI:** 10.3389/fmed.2022.840261

**Published:** 2022-02-28

**Authors:** Nouf A. Mushari, Georgios Soultanidis, Lisa Duff, Maria G. Trivieri, Zahi A. Fayad, Philip Robson, Charalampos Tsoumpas

**Affiliations:** ^1^Leeds Institute of Cardiovascular and Metabolic Medicine, University of Leeds, Leeds, United Kingdom; ^2^BioMedical Engineering and Imaging Institute, Icahn School of Medicine at Mount Sinai, New York, NY, United States; ^3^Institute of Medical and Biological Engineering, University of Leeds, Leeds, United Kingdom; ^4^Cardiovascular Institute, Icahn School of Medicine at Mount Sinai, New York, NY, United States; ^5^Department of Nuclear Medicine and Molecular Imaging, University Medical Centre Groningen, University of Groningen, Groningen, Netherlands

**Keywords:** cardiac sarcoidosis, PET-MRI, imaging, radiomics, machine learning

## Abstract

**Background:**

This study aimed to explore the radiomic features from PET images to detect active cardiac sarcoidosis (CS).

**Methods:**

Forty sarcoid patients and twenty-nine controls were scanned using FDG PET-CMR. Five feature classes were compared between the groups. From the PET images alone, two different segmentations were drawn. For segmentation A, a region of interest (ROI) was manually delineated for the patients' myocardium hot regions with standardized uptake value (SUV) higher than 2.5 and the controls' normal myocardium region. A second ROI was drawn in the entire left ventricular myocardium for both study groups, segmentation B. The conventional metrics and radiomic features were then extracted for each ROI. Mann-Whitney *U*-test and a logistic regression classifier were used to compare the individual features of the study groups.

**Results:**

For segmentation A, the SUV_min_ had the highest area under the curve (AUC) and greatest accuracy among the conventional metrics. However, for both segmentations, the AUC and accuracy of the TBR_max_ were relatively high, >0.85. Twenty-two (from segmentation A) and thirty-five (from segmentation B) of 75 radiomic features fulfilled the criteria: *P*-value < 0.00061 (after Bonferroni correction), AUC >0.5, and accuracy >0.7. Principal Component Analysis (PCA) was conducted, with five components leading to cumulative variance higher than 90%. Ten machine learning classifiers were then tested and trained. Most of them had AUCs and accuracies ≥0.8. For segmentation A, the AUCs and accuracies of all classifiers are >0.9, but k-neighbors and neural network classifiers were the highest (=1). For segmentation B, there are four classifiers with AUCs and accuracies ≥0.8. However, the gaussian process classifier indicated the highest AUC and accuracy (0.9 and 0.8, respectively).

**Conclusions:**

Radiomic analysis of the specific PET data was not proven to be necessary for the detection of CS. However, building an automated procedure will help to accelerate the analysis and potentially lead to more reproducible findings across different scanners and imaging centers and consequently improve standardization procedures that are important for clinical trials and development of more robust diagnostic protocols.

## Introduction

Sarcoidosis is a multisystem, granulomatous inflammatory disease of unknown etiology, characterized by the presence of non-caseating granulomas in the involved organs (1, 2). Sarcoidosis primarily affects the lungs. The development of this disease in the pulmonary system has been identified in more than 90% of reported cases (3, 4). However, it can affect the extrapulmonary organs as well, including the heart (5). Clinically, cardiac involvement is uncommon, manifesting in only ~5% of sarcoid patients, but it can occur without apparent symptoms, i.e., a “clinically silent” disease, which is reflected in the high rate of cardiac involvement in autopsy studies. At least 25% of patients with sarcoidosis are diagnosed with cardiac involvement (6–8).

The challenging in diagnosing cardiac sarcoidosis (CS) is due to the probability of involving any organ, leads to variability in clinical presentation (9). In addition, a lack of reliable biomarkers or diagnostic tests poses a challenge to diagnosing cardiac sarcoidosis. Furthermore, the role of advanced imaging modalities such as Cardiovascular Magnetic Resonance Imaging (CMR) with Late Gadolinium Enhancement (LGE) and [^18^F] Fluorodeoxyglucose Positron Emission Tomography [[^18^F] FDG PET] have been demonstrated in the literature to improve the identification and treatment of patients with CS. Currently, these imaging tools are critical for early diagnosis, disease prediction and progression, and therapeutic response monitoring.

To increase the diagnostic performance of [^18^F] FDG PET, it is important to suppress the use of glucose by normal cardiomyocytes as this improves its specificity. Several approaches have been proposed, including following a ketogenic diet (high fats and low carbohydrates), prolonged fasting, intravenous heparin, and usually, a combination of these methods (10). However, strategies to improve diagnostic performance do not help in up to 25% of patients, which can result in false-positive findings (11) due to failure to suppress the physiological uptake of the myocardium. A semi-quantitative analysis can be used to diagnose CS. A common tool, a maximum standardized uptake value (SUV_max_), can identify the highest uptake value within the region of interest (ROI). This can differentiate positive (CS^+^) and negative (CS^−^) results; however, in the presence of high physiological uptake, this metric fails to detect sarcoidosis within this region (12). In addition, the maximum target-to-background ratio (TBR_max_) is more robust than SUV_max_ due to the effective normalization for blood uptake (12, 13), which makes it more reliable for comparing data across patients and institutions. Radiomic features, which rely on the spatial correlations of image values or derived image-based metrics, have the potential to elucidate features robust to background physiological uptake. The purpose of this study is to explore radiomic features from PET images to identify potential candidate radiomic metrics. Specifically, this study will characterize radiomic features that separate active CS from controls.

## Materials and Methods

### Ethical Approval

This study was conducted with the approval of the Institutional Review Board at Mount Sinai (GCO # 01-1032), and all subjects gave written informed consent.

### Subject Selection

Subjects with clinical suspicion of CS based on demonstrated clinical manifestations of extracardiac lesions and/or disease were recruited at Mount Sinai Hospital in New York, to undertake a PET-CMR examination. All subjects were treatment-naïve and had to avoid carbohydrate diet for 24 h before the scan and fast during the last 12 h. The preparation for imaging followed the recent recommendations by Ishida et al. (14). After the acquisition, the results were assessed by an expert cardiologist for indications of CS and had no indications of failed suppression of FDG uptake. Subjects were divided into patients and controls based on their results. Subjects with patchy FDG uptake were designated as CS+ and were assigned to the patient group for this study (15), and those without either FDG or CMR findings were designated as control subjects for this study. Control population had normal cardiac appearance and regular echocardiography. Forty patients and twenty-nine controls met these criteria for this study. Exclusion criteria include insulin-dependent diabetes mellitus, pretest blood glucose >200 mmol/dl, menopausal phobia, pregnancy/lactation, the presence of a cardiac pacemaker or automatic implantable cardioverter-defibrillator, and renal dysfunction.

### Imaging Protocol

The simultaneous CMR with LGE and [^18^F] FDG PET on an integrated PET-CMR system (Biograph^TM^ mMR, Siemens Healthcare, Erlangen, Germany) was used in this study. Five MBq/kg of [^18^F] FDG was injected into the patients intravenously, who then waited for 10 min. Thoracic PET acquisition (one-bed position centered on the heart) took about 90 min but for this study only a late time window (last 60 min) was selected. PET images were reconstructed using the iterative ordinary Poisson ordered subset expectation maximization (OP-OSEM) with three iterations and 21 subsets on a 344 × 344 × 129 image matrix and an isotropic voxel size of 2 mm, followed by an isotropic 4 mm Gaussian post-filtering. The data obtained with PET were not respiratory-gated or ECG-gated and were not corrected for any potential motion artifacts. A 3D breath-hold Dixon-based MR image was used for attenuation correction. Simultaneously with PET imaging, CMR was performed with electrocardiograph triggered; the scan included short-axis T2 mapping and cine images. Approximately 15 min after 0.2 mmol/kg gadolinium injection, inversion-recovery fast gradient-echo LGE sequences were acquired.

### Segmentations

3D slicer software (Version 4.11.2; https://www.slicer.org) was used for the segmentation (16, 17). Segmentations were performed by study personnel according to methods used in a previous study (12).

#### Segmentation A

From the PET images (with use of CMR for anatomical localization, and aiding in focal lesion identification when possible) of the patient group, an ROI was manually drawn in the hot region of the myocardium with an SUV higher than 2.5, which is a cut-off value previously used to differentiate between benign (normal in cases of CS) and malignant (abnormal in cases of CS) lesions (18, 19). For patients with more than one focal lesion, the largest and most active was selected. Due to the focal nature of the disease, applying a threshold helped ensure that the extracted features are only from voxels with abnormal uptakes. For the control group, an ROI was drawn manually in the normal myocardium. Once the SUV_max_ and SUV_mean_ (in the blood pool of the right atrium) were extracted, the TBR_max_ was calculated using the following equation:


$$\begin{array}{l} {\text{TBR}}_{\max}=\frac{{\text{SUV}}_{\max}\;\left(\text{target}\right)}{{\text{SUV}}_{\text{mean}}\;\left(\text{background}\right)} \end{array}$$

Thirty-five subjects out of forty who had a TBR_max_ within the range of 1 to 3 and patchy uptake were labeled as patients. The remaining five subjects who had TBR_max_ > 3 were excluded as failed suppression could not be completely discounted in these cases (12) even though the FDG was patchy and initially included in the study cohort and subsequently in the study cohort for segmentation B.

#### Segmentation B

As the approach A took into account both intensity and pattern, it was useful to investigate a different approach that was independent of these. From the PET images, an ROI was drawn in the entire left ventricular myocardium for forty patients and twenty-nine controls regardless of the TBR_max_ findings and SUV thresholds to compare the reliability of features among segmentation approaches. Radiomic features and conventional metrics were then extracted.

### Feature Extraction

PyRadiomics (Version 3.0.1) was used to extract five feature classes (75 features in total) from the PET image ROIs of the patients and controls (20) in addition to the conventional metrics (7 metrics). PyRadiomics adheres to the image biomarker standardization initiative (IBSI's feature definitions). A bin width of 0.05 was applied. All other parameters were left as default. Harmonization was not required for these datasets as they originated from a single scanner. A list of all radiomic features and conventional metrics is shown in Supplementary Material 1.

### Statistical Analysis

Statistical analyses were undertaken using Scikit-learn software (Version 0.23.2) (21). Mann–Whitney *U*-test was used to compare the radiomic features of the study groups. The *P*-value was adjusted using a Bonferroni correction approach for multiple tests [*P*-value (0.05) divided by the number of features (82)] and the corrected *P*-value of < 0.00061 was considered to be statistically significant. Logistic regression classifiers were then trained with individual features. Stratified 5-fold cross-validation was used to determine the mean area under the curve (AUC), mean accuracy, and 95% confidence intervals (CIs). Features with a *P*-value < 0.00061, AUC >0.5, and accuracy >0.7 were retained. In addition, principal component analysis (PCA) was used to identify highly correlated features and reduce feature redundancy. PCA reduces a large number of features into a small number of principal components (PCs). Components that explained 90% of the cumulative variance were retained. Lastly, to find the best machine learning (ML) algorithm, PCs were used as an input to test and train the following ten classifiers: Random Forest, Logistic Regression, Support Vector Machine, Decision Tree, Gaussian Process Classifier, Stochastic Gradient Descent, Perceptron Classifier, Passive Aggressive Classifier, Neural Network Classifier and K-neighbors Classifier with stratified 5-fold cross-validation.

## Results

### Conventional Metrics Diagnostic Utility

**Figure 1:**
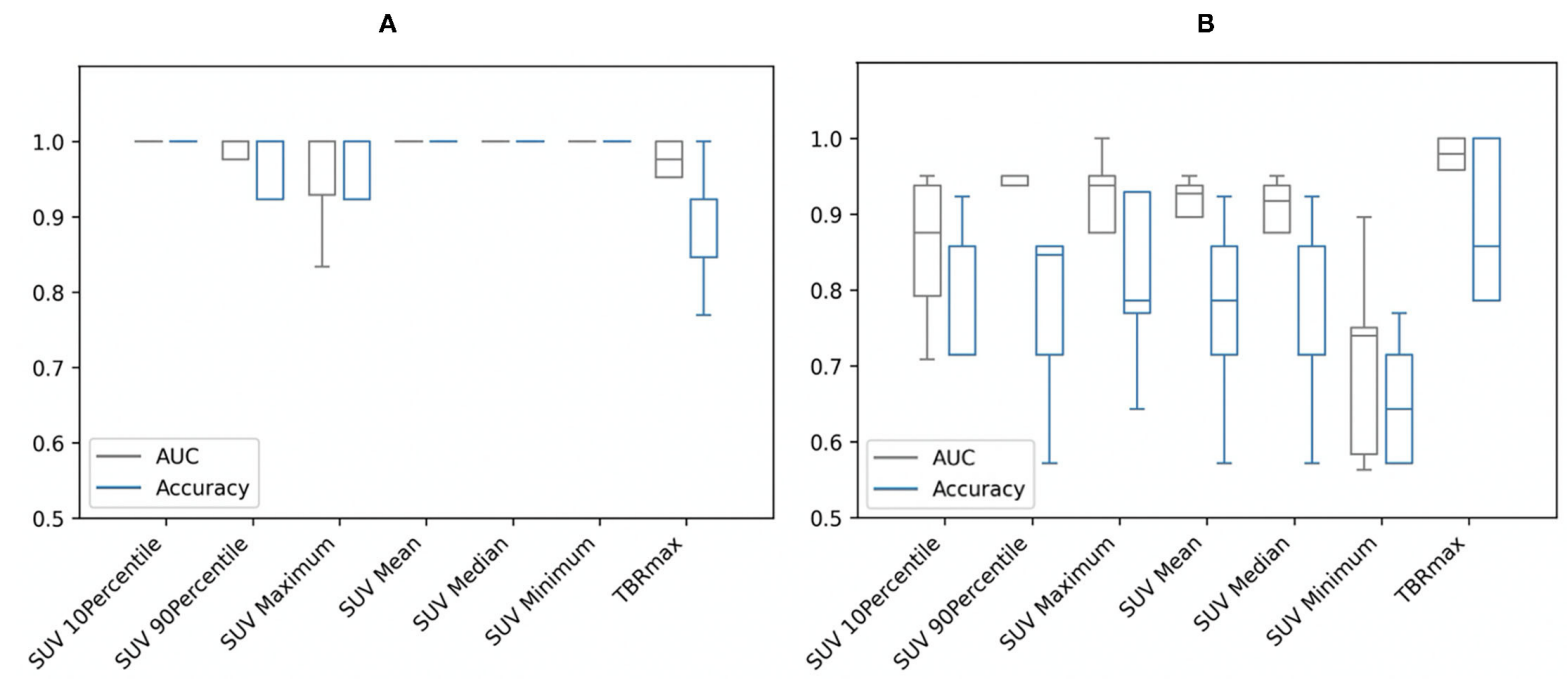
Area under the curve (AUC) and accuracy with stratified 5-fold cross-validation of the conventional metrics of **(A)** segmentation A and **(B)** segmentation B. SUV, standardized uptake value; TBRmax, maximum target-to-background ratio.

The results are relatively different by applying the Mann–Whitney *U*-tests on the conventional metrics of the different study groups for each segmentation separately. Predictably, for segmentation A, the SUV_min_ had the highest AUC and greatest accuracy due to specifying SUV >2.5 as the minimum value for the patient group, while for segmentation B, the highest performance was for TBR_max_ (see Figure 1). However, for both segmentations, the AUC and accuracy of the TBR_max_ were relatively high and had similar results regardless of the segmentation approach (AUC 0.96; accuracy 0.88–0.89 for segmentation A & B, respectively). This slight difference in TBR_max_ results between both segmentations came from the difference in the number of participants in the patient group who met the criteria for each segmentation.

### Individual Radiomic Features Diagnostic Utility

**Figure 2:**
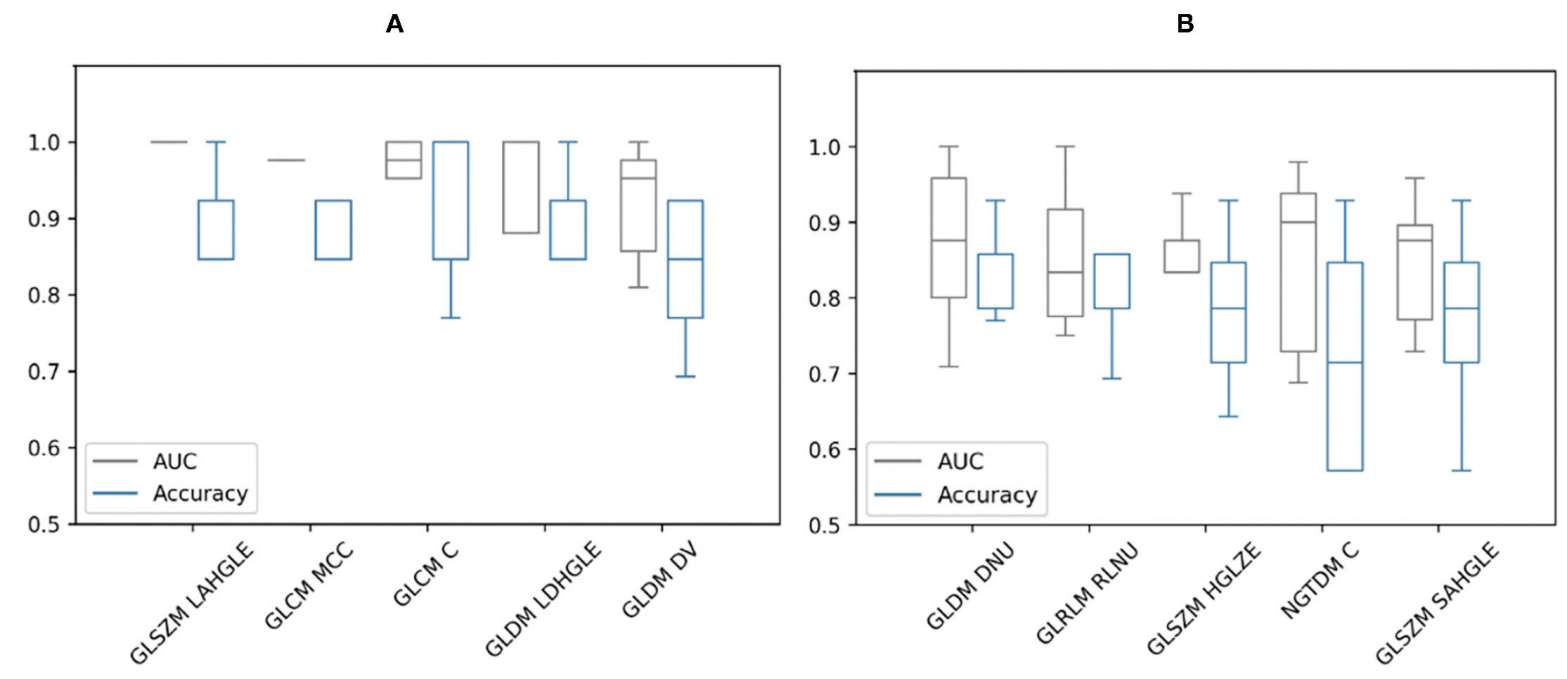
Area under the curve (AUC) and accuracy with stratified 5-fold cross-validation of the five best-performing radiomic features of **(A)** segmentation A and **(B)** segmentation B based on AUC values. GLSZM, Gray Level Size Zone Matrix; LAHGLE, Large Area High Gray Level Emphasis; GLCM, Gray Level Co-occurrence Matrix; MCC, Maximal Correlation Coefficient; GLCM C, Correlation; GLDM, Gray Level Dependence Matrix; LDHGLE, Large Dependence High Gray Level Emphasis; DV, Dependence Variance; DNU, Dependence Non-Uniformity; GLRLM, Gray Level Run Length Matrix; RLNU, Run Length Non-Uniformity; HGLZE, High Gray Level Zone Emphasis; NGTDM, Neighboring Gray Tone Difference Matrix; NGTDM C, Complexity; SAHGLE, Small Area High Gray Level Emphasis.

From the Mann–Whitney *U*-tests, for segmentation A: 40 of the 75 radiomic features and for segmentation B: 61 of the 75 showed statistically significant differences between patients and controls, with a *P*-value < 0.00061. The five best radiomic features based on *P*-values for both segmentations are shown in Table 1. After applying a logistic regression classifier, only 22 radiomic features for segmentation A and 35 radiomic features for segmentation B fulfilled the following criteria: *P*-value < 0.00061, AUC >0.5, and accuracy >0.7. The AUC and accuracy (95% CI for each criterion) with stratified 5-fold cross-validation of the five best-performing radiomic features based on the AUC value are shown in Figure 2. All values of radiomic features and conventional metrics for both segmentations are provided in Supplementary Material 2.

**Table 1:** Conventional metrics and five best performing radiomic features for the different segmentations based on *P*-values.

	**Segmentation A**			**Segmentation B**		
	**Feature**	***P*-value**	**AUC**	**Feature**	***P*-value**	**AUC**
Conventional	SUV 10 percentile	1 × 10^−11^	0.99	SUV 10 percentile	6 × 10^−7^	0.85
	SUV 90 percentile	1 × 10^−10^	0.96	SUV 90 percentile	3 × 10^−8^	0.90
	SUV maximum	3 × 10^−10^	0.95	SUV maximum	8 × 10^−9^	0.90
	SUV mean	1 × 10^−10^	0.97	SUV mean	6 × 10^−8^	0.88
	SUV median	1 × 10^−10^	0.97	SUV median	2 × 10^−7^	0.88
	SUV minimum	6 × 10^−13^	1.00	SUV minimum	9 × 10^−3^	0.71
	TBR_max_	1 × 10^−10^	0.96	TBR_max_	3 × 10^−11^	0.96
Radiomics	GLDM_small dependence low gray level emphasis	3 × 10^−13^	1.00	GLSZM_low gray level zone emphasis	5 × 10^−8^	0.85
	GLCM_inverse difference normalized	1 × 10^−11^	1.00	GLDM_dependence non-uniformity	1 × 10^−7^	0.87
	GLSZM_small area low gray level emphasis	1 ×10^−11^	0.99	NGTDM_complexity	1 ×10^−7^	0.85
	GLSZM_large area high gray level emphasis	3 ×10^−11^	1.00	GLSZM_high gray level zone emphasis	1 ×10^−7^	0.85
	GLCM_maximal correlation coefficient	5 ×10^−11^	0.98	GLSZM_small area high gray level emphasis	1 ×10^−7^	0.85

*SUV, Standardized Uptake Value; TBR_max_, maximum Target-to-Background Ratio; GLDM, Gray Level Dependence Matrix; GLCM, Gray Level Co-occurrence Matrix; GLSZM, Gray Level Size Zone Matrix; NGTDM, Neighboring Gray Tone Difference Matrix*.

### Principal Component Analysis and Machine Learning

**Figure 3:**
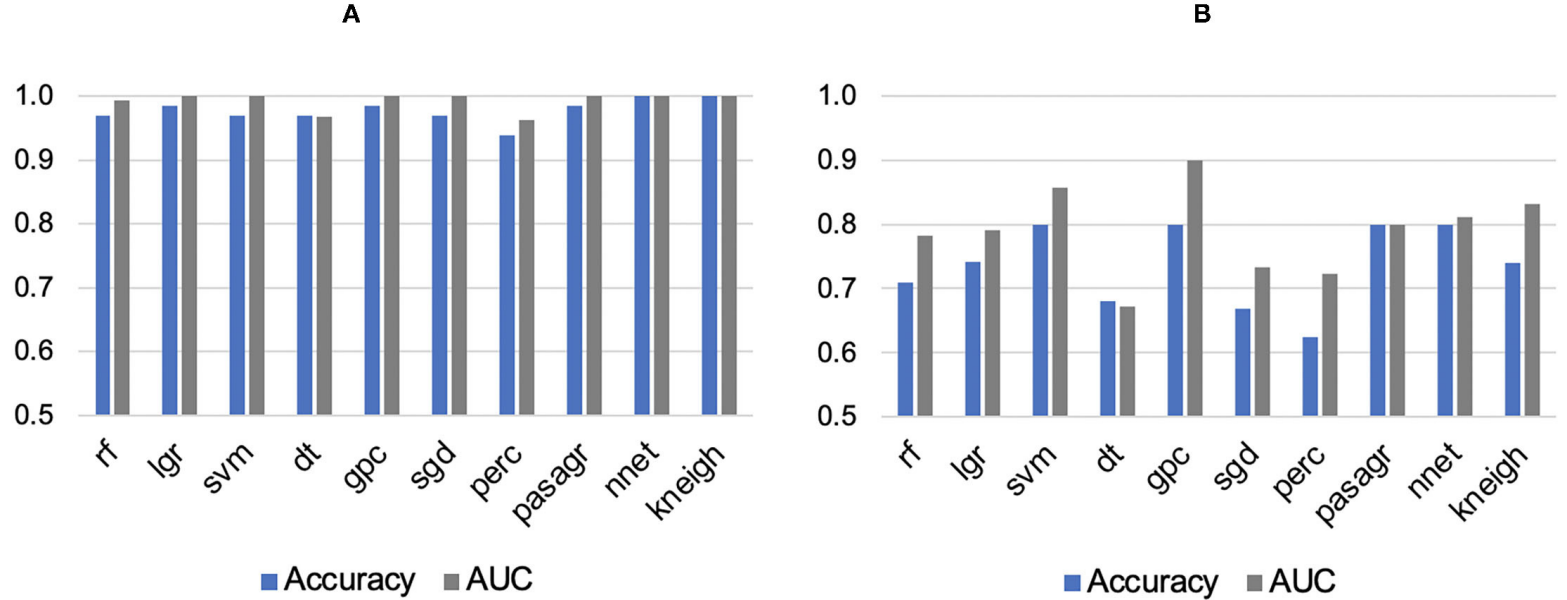
Areas under the curve (AUC) and accuracies of machine learning classifiers for **(A)** Segmentation A and **(B)** Segmentation B. rf, Random Forest; lgr, Logistic Regression; svm, Support Vector Machine; dt, Decision Tree; gpc, Gaussian Process Classifier; sgd, Stochastic Gradient Descent; perc, Perceptron Classifier; pasagr, Passive Aggressive Classifier; nnet, Neural Network Classifier; kneigh, K-neighbors Classifier.

**Figure 4:**
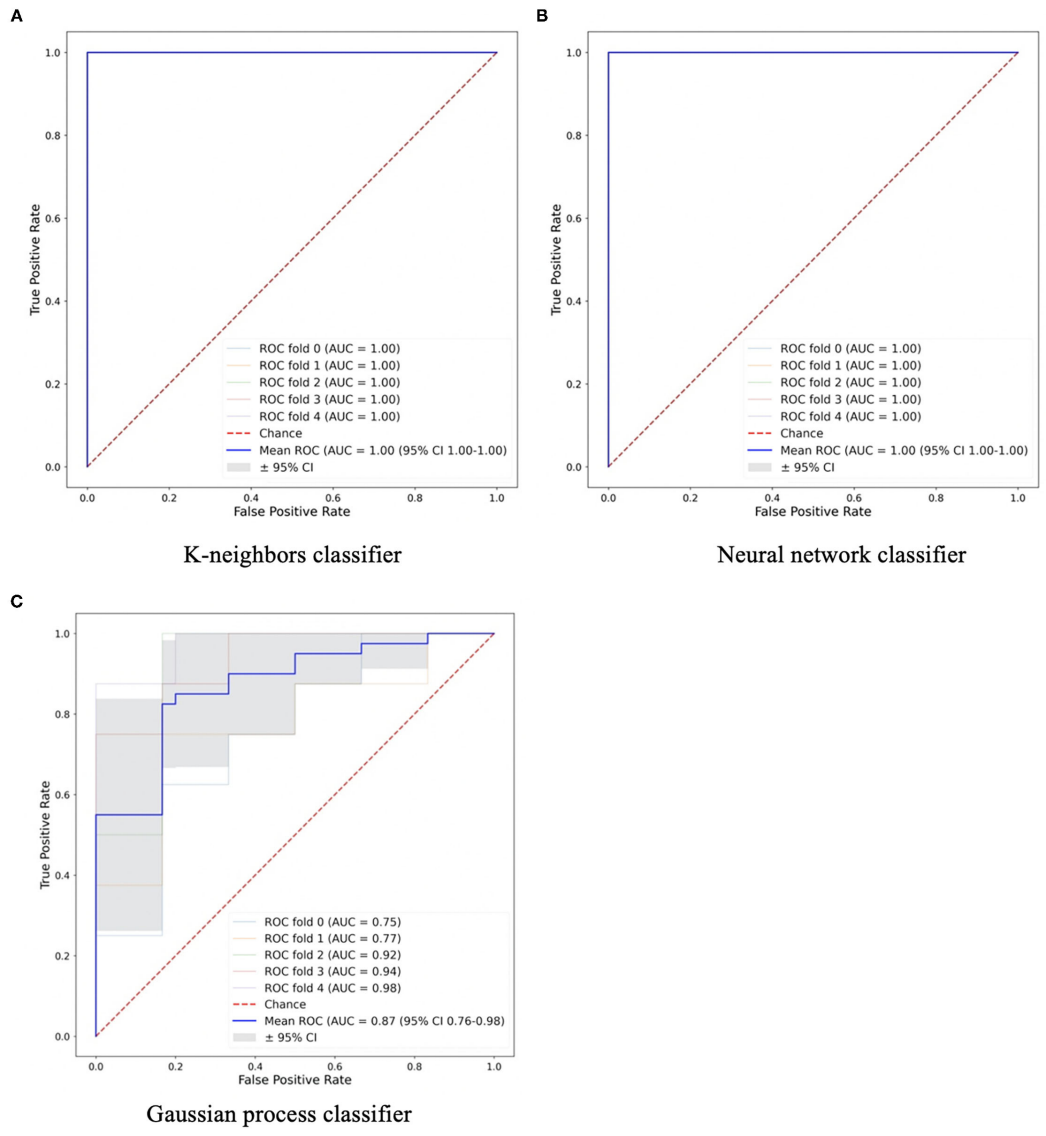
The machine learning classifiers with high performance in **(A,B)** Segmentation A and **(C)** Segmentation B.

As the SUV-related metrics tend to overperform, and to study the performance of non-first order features, the SUV-related metrics were excluded from the PCA. By applying PCA, five PCs were retained to explain 90% of the information. These PCs were used to test and train the ML classifiers. Most of them had AUCs and accuracies ≥0.8. For segmentation A, all classifiers showed high performance in terms of AUC (95% CI 0.88–1.00) and accuracy (95% CI 0.87–1.00), with values >0.9. A k-neighbors and neural network classifiers showed the highest AUC and greatest accuracy, with values equal to 1.00, as shown in Figure 3. For segmentation B, there are four classifiers with AUCs and accuracies ≥0.8, Figure 3. However, the gaussian process classifier indicated the highest AUC and accuracy (0.9 and 0.8, respectively). The ROC curves of the k-neighbors, neural network, and gaussian process classifiers are shown in Figure 4. The actual values of Figures 2, 3 are provided in Supplementary Material 3.

## Discussion

This study aimed to explore the diagnostic utility of radiomic features compared to conventional metrics to distinguish between study groups and find the best performance ML classifier to create an automated model. From segmentation A, some conventional metrics like SUV_min_ showed high performance individually. These results were predictable as they are affected by the distribution of voxel intensities within the ROI, one of the criteria for including the patients at the first place. In addition, these features cannot be relied upon because they are greatly affected by the success of glucose suppression in normal cardiomyocytes. TBR_max_ was the most reliable metric over other conventional metrics among both segmentations. Although the TBR_max_ is sensitive to noise and it is not necessarily easy to harmonize across different scanners and imaging centers, types of data, and parameters, this is not the case in this study as datasets originated from a single scanner and institution. Therefore, when comparing TBR_max_ with those of the five-best performance radiomic features, the superiority of TBR_max_ over the rest of the features can be clearly seen. This outcome supports any previous studies that utilized TBR_max_.

From segmentation A, by comparing the diagnostic utility of individual radiomic features, GLSZM-Large Area High Gray Level Emphasis radiomic feature showed the best performance in terms of AUC and accuracy. This feature measures the proportion in the image of the joint distribution of larger size zones with higher gray level values. This means there is a difference in gray level zones between patients and controls. However, it cannot be reliable due to the criteria of this segmentation approach that is based on SUV threshold and TBR_max_. On the other hand, from segmentation B, the best performing radiomic feature was GLDM_Dependence Non-Uniformity with AUC (0.87) and accuracy (0.83). This feature measures the heterogeneity in the ROIs. The values of this feature are higher in sarcoid patients than controls which illustrates more heterogeneous regions in the group of patients. In addition, many other features measure heterogeneity with high AUCs and accuracies. These features look at the spatial relationships rather than voxels values themselves. However, these features had large error bars, unlike the TBR_max_ which had very small bars regardless of the segmentation approach.

Several studies of different diseases advocated the importance of radiomic analysis to predict outcomes (22, 23). However, the findings across these studies are not replicated; instead, they are conflicted. Technical issues may illustrate this difference in results among studies, such as ROI size, scanner resolution, reconstruction, and segmentation algorithms, or any other unrevealed factors. High scanner resolution and large number of voxels can affect some radiomic features by increasing their values (24). In terms of segmentation algorithms, numerous studies indicated that using different segmentation methods gave close results in survival analyses (23, 25). In addition, Cheng et al. (23) argued that no significant difference exists between radiomic features when using different segmentation methods, unlike SUV_max_ and SUV_mean_. They reported, in addition, that the effect of utilizing different attenuation correction methods on radiomic features was not significant. At the same time Yip et al. (26) had contrasting results, as some of the features were affected by the attenuation correction method. However, in this study, there was a clear difference between radiomic features when using different segmentation approaches. This may be due to the different sizes of ROIs and the voxel intensities included in each segmentation. Applying the approach of segmentation A, it can provide a good differentiation between study groups based on the conventional metrics such as SUV_min_ and TBR_max_. However, this approach can be influenced by observer experience, especially for cases with very small hotspots. Conversely, segmentation B approach is more robust and efficient.

This study is subject to some limitations. First, the sample size is relatively small, and more extensive studies are needed to confirm these results. This is of great significance to prevent overfitting and type I errors. Applying a Bonferroni correction and dimensionality reduction techniques resulted in reducing the effect of this issue. In addition, the lack of an automated segmentation, a segmentation reference to compare with, unavailability of an independent clinical gold standard to validate the performance of the model that was trained on initial input data are other limitations for this study. In addition, the selection of only one focal lesion per patient in segmentation A was considered a limitation of this approach. Furthermore, the models proposed in this study should be validated in normal controls showing non-specific physiological uptake. This study showed uncertainty results of radiomic features and expanding the study to test the reproducibility of the results is required. New knowledge gained from this study is that using radiomic analysis does not provide any additional information related to disease activity in these patients. However, building an automated model regardless of the strategies used for glucose suppression and/or observer experience may prove helpful in further studies. Furthermore, in this study, the MRI acquisitions were not utilized, except for providing anatomical information. In this study the main goal was the radiomic features on PET; the designated tool for CS.

## Conclusion

Radiomic analysis of PET data may not be a useful approach to detect CS. Several radiomic features that were not related to first-order tracer uptake showed high AUC and accuracy with *P*-value < 0.00061. However, by measuring AUCs and accuracies, large error bars can weaken the results. TBR_max_ showed its superiority over all other conventional and radiomic features in both segmentation approaches. This methodology needs to be validated further in normal control subjects showing non-specific physiological uptake.

## Data Availability Statement

The original contributions presented in the study are included in the article/Supplementary Material, further inquiries can be directed to the corresponding author/s.

## Ethics Statement

This study was conducted with the approval of the Institutional Review Board at Mount Sinai. The patients/participants provided their written informed consent to participate in this study.

## Author Contributions

NM segmented all the datasets using 3D slicer software, analyzed the data using PyRadiomics, performed the statistical analysis, analyzed the results, and wrote the manuscript. GS shared datasets, reviewed segmentations, and helped in modifying code as well as in the guidance of the project. LD wrote python code and helped to modify it and provide essential guidance on how to perform the optimization of the radiomic analysis, and machine learning approaches. MT facilitated the availability of data. MT, ZF, and PR contributed to reviewing the manuscript and the overall guidance of the project and data. ZF is the PI of the NIH grant. CT supervised the specific study and helped in restructuring and reviewing the manuscript. All authors contributed to the article and approved the submitted version.

## Funding

NM is fully funded by Taif University, Saudi Arabia. GS and PR are supported by NIH grant R01HL071021. LD is fully funded by the EPSRC Centre for Doctoral Training in Tissue Engineering and Regenerative Medicine: Innovation in Medical and Biological Engineering – grant number EP/L014823/1.

## Conflict of Interest

The authors declare that the research was conducted in the absence of any commercial or financial relationships that could be construed as a potential conflict of interest.

## Publisher's Note

All claims expressed in this article are solely those of the authors and do not necessarily represent those of their affiliated organizations, or those of the publisher, the editors and the reviewers. Any product that may be evaluated in this article, or claim that may be made by its manufacturer, is not guaranteed or endorsed by the publisher.

## Abbreviations

CS: cardiac sarcoidosis
SUV: standardized uptake value
[^18^F]FDG PET: [^18^F]-fluorodeoxyglucose positron emission tomography
CMR: cardiovascular magnetic resonance imaging
AUC: area under the curve
PCA: principal component analysis.

## References

[1] Birnie DH Nery PB Ha AC Beanlands RS. Cardiac sarcoidosis. J Am Coll Cardiol. (2016) 68:411–21. 10.1016/j.jacc.2016.03.605 27443438

[2] Hulten E Aslam S Osborne M Abbasi S Bittencourt MS Blankstein R. Cardiac sarcoidosis—state of the art review. Cardiovasc Diag Therapy. (2016) 6:50. 10.3978/j.issn.2223-3652.2015.12.13 26885492 PMC4731586

[3] Ginelliová A Farkaš D Iannaccone SF Vyhnálková V. Sudden unexpected death due to severe pulmonary and cardiac sarcoidosis. Forensic Sci Med Pathol. (2016) 12:319–23. 10.1007/s12024-016-9792-y 27379608

[4] Petek BJ Rosenthal DG Patton KK Behnia S Keller JM Collins BF. Cardiac sarcoidosis: diagnosis confirmation by bronchoalveolar lavage and lung biopsy. Res Med. (2018) 144:S13–S9. 10.1016/j.rmed.2018.09.008 30249376

[5] Deng JC Baughman RP Lynch Iii JP editors. Cardiac Involvement in Sarcoidosis. Seminars in Respiratory and Critical Care Medicine. Copyright© 2002 by Thieme Medical Publishers, Inc., 333 Seventh Avenue, New (2002). 10.1055/s-2002-36516 16088647

[6] Iwai K Tachibana T Takemura T Matsui Y Kitalchi M Kawabata Y. Pathological studies on sarcoidosis autopsy. I. Epidemiological features of 320 cases in Japan. Pathol Int. (1993) 43:372–6. 10.1111/j.1440-1827.1993.tb01148.x 8372682

[7] Perry A Vuitch F. Causes of death in patients with sarcoidosis. A morphologic study of 38 autopsies with clinicopathologic correlations. Arch Pathol Lab Med. (1995) 119:167. 7848065

[8] Kim JS Judson MA Donnino R Gold M Cooper Jr LT Prystowsky EN. Cardiac sarcoidosis. Am Heart J. (2009) 157:9–21. 10.1016/j.ahj.2008.09.009 19081391

[9] Trivieri MG Spagnolo P Birnie D Liu P Drake W Kovacic JC. Challenges in cardiac and pulmonary sarcoidosis: JACC state-of-the-art review. J Am Coll Cardiol. (2020) 76:1878–901. 10.1016/j.jacc.2020.08.042 33059834 PMC7808240

[10] Chareonthaitawee P Beanlands RS Chen W Dorbala S Miller EJ Murthy VL. Joint SNMMI–ASNC expert consensus document on the role of 18F-FDG PET/CT in cardiac sarcoid detection and therapy monitoring. J Nucl Med. (2017) 58:1341–53. 10.2967/jnumed.117.196287 28765228 PMC6944184

[11] Osborne MT Hulten EA Murthy VL Skali H Taqueti VR Dorbala S. Patient preparation for cardiac fluorine-18 fluorodeoxyglucose positron emission tomography imaging of inflammation. J Nucl Cardiol. (2017) 24:86–99. 10.1007/s12350-016-0502-7 27277502 PMC5841447

[12] Dweck MR Abgral R Trivieri MG Robson PM Karakatsanis N Mani V. Hybrid magnetic resonance imaging and positron emission tomography with fluorodeoxyglucose to diagnose active cardiac sarcoidosis. JACC. (2018) 11:94–107. 10.1016/j.jcmg.2017.02.021 28624396 PMC5995315

[13] Chen W Dilsizian V. PET assessment of vascular inflammation and atherosclerotic plaques: SUV or TBR? J Nucl Med. (2015) 56:503–4. 10.2967/jnumed.115.154385 25722451

[14] Ishida Y Yoshinaga K Miyagawa M Moroi M Kondoh C Kiso K. Recommendations for 18 F-fluorodeoxyglucose positron emission tomography imaging for cardiac sarcoidosis: Japanese society of nuclear cardiology recommendations. Ann Nucl Med. (2014) 28:393–403. 10.1007/s12149-014-0806-0 24464391

[15] Birnie DH Sauer WH Bogun F Cooper JM Culver DA Duvernoy CS. HRS expert consensus statement on the diagnosis and management of arrhythmias associated with cardiac sarcoidosis. Heart Rhythm. (2014) 11:1304–23. 10.1016/j.hrthm.2014.03.043 24819193

[16] Fedorov A Beichel R Kalpathy-Cramer J Finet J Fillion-Robin J-C Pujol S. 3D Slicer as an image computing platform for the Quantitative Imaging Network. Magn Res Imag. (2012) 30:1323–41. 10.1016/j.mri.2012.05.001 22770690 PMC3466397

[17] Kikinis R Pieper SD Vosburgh KG. 3D Slicer: A Platform for Subject-Specific Image Analysis, Visualization, and Clinical Support. Intraoperative Imaging and Image-Guided Therapy. New York, NY: Springer (2014). p. 277–89.

[18] Kadaria D Freire AX SultanAli I Zaman MK Archie DS Weiman DS. Dual time point positron emission tomography/computed tomography scan in evaluation of intrathoracic lesions in an area endemic for histoplasmosis and with high prevalence of sarcoidosis. Am J Med Sci. (2013) 346:358–62. 10.1097/MAJ.0b013e31827b9b6d 23276900

[19] Wang T Sun H Guo Y Zou L. 18F-FDG PET/CT quantitative parameters and texture analysis effectively differentiate endometrial precancerous lesion and early-stage carcinoma. Mol Imag. (2019) 18:1536012119856965. 10.1177/1536012119856965 31198089 PMC6572902

[20] Van Griethuysen JJ Fedorov A Parmar C Hosny A Aucoin N Narayan V. Computational radiomics system to decode the radiographic phenotype. Can Res. (2017) 77:e104–7. 10.1158/0008-5472.CAN-17-0339 29092951 PMC5672828

[21] Pedregosa F Varoquaux G Gramfort A Michel V Thirion B Grisel O. Scikit-learn: machine learning in python. J Mach Learn Res. (2011) 12:2825–30.

[22] Apostolova I Ego K Steffen IG Buchert R Wertzel H Achenbach HJ. The asphericity of the metabolic tumour volume in NSCLC: correlation with histopathology and molecular markers. Eur J Nucl Med Mol Imag. (2016) 43:2360–73. 10.1007/s00259-016-3452-z 27470327

[23] Cheng N-M Fang Y-HD Tsan D-L Hsu C-H Yen T-C. Respiration-averaged CT for attenuation correction of PET images–impact on PET texture features in non-small cell lung cancer patients. PLoS ONE. (2016) 11:e0150509. 10.1371/journal.pone.0150509 26930211 PMC4773107

[24] Han S Woo S Suh CH Kim YJ Oh JS Lee JJ. A systematic review of the prognostic value of texture analysis in 18 F-FDG PET in lung cancer. Ann Nucl Med. (2018) 32:602–10. 10.1007/s12149-018-1281-9 30014440

[25] Bashir U Azad G Siddique MM Dhillon S Patel N Bassett P. The effects of segmentation algorithms on the measurement of 18 F-FDG PET texture parameters in non-small cell lung cancer. Ejnmmi Res. (2017) 7:60. 10.1186/s13550-017-0310-3 28748524 PMC5529305

[26] Yip S McCall K Aristophanous M Chen AB Aerts HJ Berbeco R. Comparison of texture features derived from static and respiratory-gated PET images in non-small cell lung cancer. PLoS ONE. (2014) 9:e115510. 10.1371/journal.pone.0115510 25517987 PMC4269460

