# Supplementary material for: Exploring the Utility of Radiomic Feature Extraction to Improve the Diagnostic Accuracy of Cardiac Sarcoidosis Using FDG PET
Source: Front Med (Lausanne). 2022 Feb 28;9:840261. doi: 10.3389/fmed.2022.840261 (PMC8920041; doi:10.3389/fmed.2022.840261)
Supplement: Supplementary file 3 [file Data_Sheet_3.docx]

Supplementary Material 3

Table 1 Area under the curve (AUC) and accuracy of the five best-performing radiomic features for the different segmentations based on AUC values. GLSZM: Gray Level Size Zone Matrix, LAHGLE: Large Area High Gray Level Emphasis, GLCM: Gray Level Co-occurrence Matrix, MCC: Maximal Correlation Coefficient, GLCM_C: Correlation, GLDM: Gray Level Dependence Matrix, LDHGLE: Large Dependence High Gray Level Emphasis, DV: Dependence Variance, DNU: Dependence Non-Uniformity, GLRLM: Gray Level Run Length Matrix, RLNU: Run Length Non-Uniformity, HGLZE: High Gray Level Zone Emphasis, NGTDM: Neighboring Gray Tone Difference Matrix, NGTDM_C: Complexity, SAHGLE: Small Area High Gray Level Emphasis.

| **Segmentation A** | | | **Segmentation B** | | |
| --- | --- | --- | --- | --- | --- |
| **Feature** | **Accuracy** | **AUC** | **Feature** | **Accuracy** | **AUC** |
| GLSZM_LAHGLE | 0.91 | 1.00 | GLDM_DNU | 0.83 | 0.87 |
| GLCM_MCC | 0.88 | 0.98 | GLRLM_RLNU | 0.81 | 0.86 |
| GLCM_C | 0.89 | 0.96 | GLSZM_HGLZE | 0.78 | 0.85 |
| GLDM_LDHGLE | 0.86 | 0.95 | NGTDM_C | 0.73 | 0.85 |
| GLDM_DV | 0.83 | 0.92 | GLSZM_SAHGLE | 0.77 | 0.85 |

Table 2 Area under the curve (AUC) and accuracy of the machine learning classifiers for the different segmentations.

| **Machine Learning Classifier** | **Segmentation A** | | **Segmentation B** | |
| --- | --- | --- | --- | --- |
|  | **Accuracy** | **AUC** | **Accuracy** | **AUC** |
| Random Forest | 0.97 | 0.99 | 0.71 | 0.78 |
| Logistic Regression | 0.99 | 1.00 | 0.74 | 0.79 |
| Support Vector Machine | 0.97 | 1.00 | 0.80 | 0.86 |
| Decision Tree | 0.97 | 0.97 | 0.68 | 0.67 |
| Gaussian Process Classifier | 0.99 | 1.00 | 0.80 | 0.90 |
| Stochastic Gradient Descent | 0.97 | 1.00 | 0.67 | 0.73 |
| Perceptron Classifier | 0.94 | 0.96 | 0.63 | 0.72 |
| Passive Aggressive Classifier | 0.99 | 1.00 | 0.80 | 0.80 |
| Neural Network Classifier | 1.00 | 1.00 | 0.80 | 0.81 |
| K-neighbors Classifier | 1.00 | 1.00 | 0.74 | 0.83 |
